# Drug-Induced Pseudoporphyria: A Case Report

**DOI:** 10.7759/cureus.57574

**Published:** 2024-04-04

**Authors:** Shivani D Jangid, Vikrant Saoji, Bhushan Madke, Drishti M Bhatt

**Affiliations:** 1 Dermatology, Jawaharlal Nehru Medical College, Datta Meghe Institute of Higher Education and Research, Wardha, IND

**Keywords:** wood’s lamp, non-steroidal anti-inflammatory drugs (nsaids), bullous dermatoses, photosensitivity disorders, pseudoporphyria

## Abstract

Pseudoporphyria is an uncommon dermatosis resembling porphyria cutanea tarda (PCT). The exclusion of true porphyria, especially PCT, is critically essential for diagnosing pseudoporphyria. It has an unknown underlying pathophysiology with a normal or near-normal porphyrin profile. Pseudoporphyria has been associated with chronic renal failure and hemodialysis, medications, and tanning beds. In drug-induced pseudoporphyria cases, eliminating the suspected photosensitizing drug improves the disease typically within weeks to months (on average eight weeks). In genetically predisposed individuals, phototoxic metabolites may trigger the development of skin fragility, bullae, milia, and scarring on the dorsum of the hands and other sun-exposed areas. Wearing a broad-spectrum sunscreen and maintaining strict ultraviolet protection is essential in cases of pseudoporphyria.

We report the case of a 20-year-old male who presented to us with complaints of photosensitivity and multiple erosions with irregular scars over photo-exposed areas involving the dorsum of the hands and face predominantly. The patient was evaluated further to determine the underlying cause. A wood’s lamp examination of the urine was done, which did not show fluorescence. Based on clinical and laboratory findings, the diagnosis of pseudoporphyria was made, and the patient was started on the oral antimalarial agent hydroxychloroquine sulfate with strict sun protection.

## Introduction

Pseudoporphyria has also been referred to as “drug-induced pseudoporphyria cutanea tarda,” “drug-induced pseudoporphyria,” “bullous dermatosis of hemodialysis,” “pseudoporphyria cutanea tarda,” and “bullous dermatosis in end-stage renal failure.” Sun-exposed skin develops vesicles, bullae, skin fragility, milia, and scarring. The dorsum of the hands is typically affected. However, fingers, extensor legs, upper chest, or face may also be impacted [1].

Pseudoporphyria is a photodistributed bullous syndrome that shares clinical and histological similarities with porphyria cutanea tarda (PCT) without concomitant biochemical porphyrin abnormalities. Multiple etiological factors, such as medications, genetic predisposition, exposure to high ultraviolet (UV) radiation, and chronic renal failure, have been linked to its pathogenesis [2]. Several photosensitizing drugs, hormone replacement therapy, ultraviolet A (UVA) radiation from tanning beds, hepatitis C, sarcoidosis, Sjögren’s syndrome, hepatoma, human immunodeficiency virus infection, and lupus erythematosus are linked to pseudoporphyria. Naproxen, tetracycline, fluoroquinolones, voriconazole, furosemide, chlorthalidone, butamide, hydrochlorothiazide/triamterene, amiodarone, and cyclosporine are medications that are frequently linked to pseudoporphyria [3].

The only treatments available are photoprotection and the suspension of any potential medication [4]. The exact process via which some medications, UV light, or hemodialysis cause pseudoporphyria remains unknown. In the case of a large number of non-steroidal anti-inflammatory drugs (NSAIDs), a phototoxic mechanism has been proposed. Similar to porphyrin structures, the heterobicyclic or naphthalene parts of naproxen, napumetone, ketoprofen, a piroxicam metabolite, and tiaprofenic acid absorb UV radiation at wavelengths longer than 310 nm. Singlet oxygen is produced by naproxen and other NSAIDs when they come into contact with UVA rays. This singlet oxygen could then cause phototoxicity [5]. Dialysis may hasten the development of pseudoporphyria in genetically susceptible patients by inducing oxidative stress or by signaling a more advanced degree of renal failure [6].

## Case presentation

**Figure 1:**
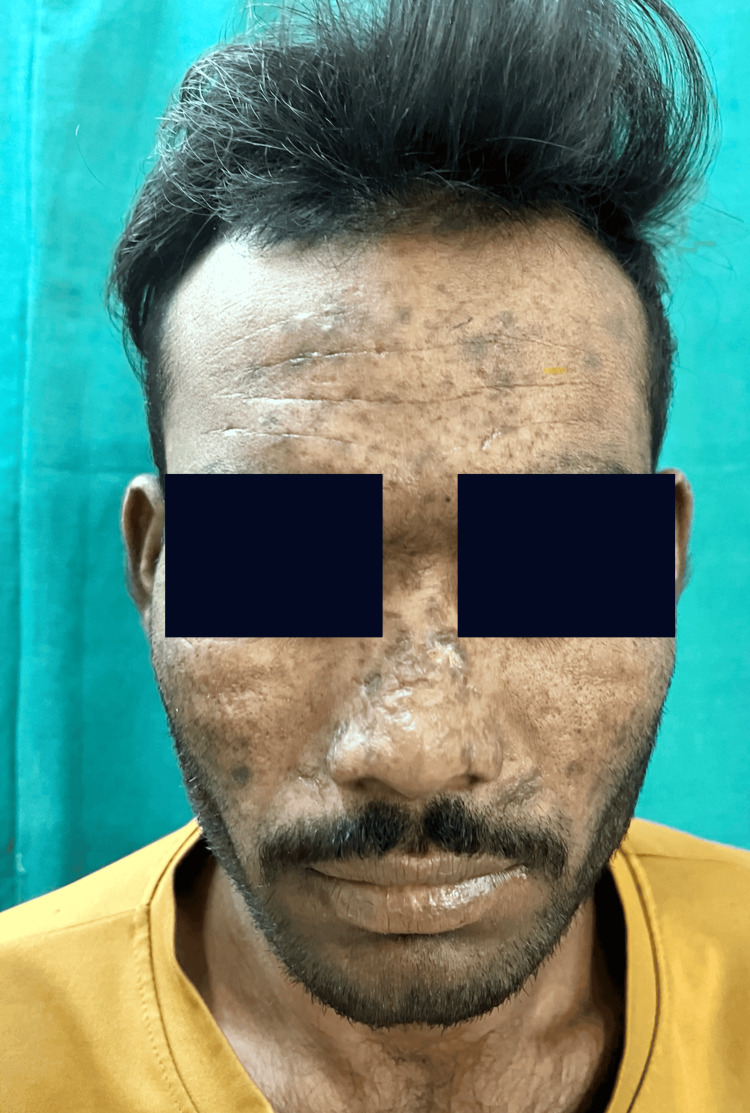
Post-inflammatory hyperpigmentation with multiple irregular scars.

**Figure 2:**
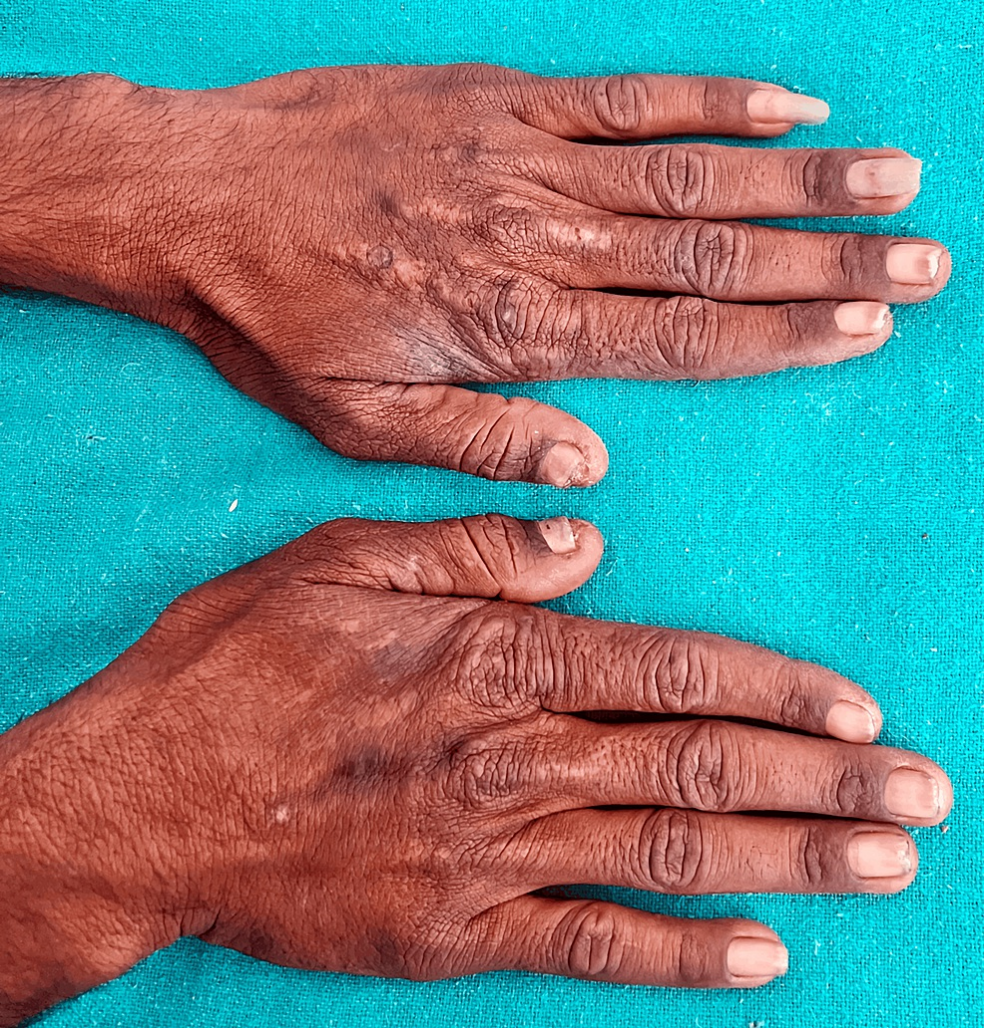
Multiple erosions with scarring and post-inflammatory hyperpigmentation.

**Figure 3:**
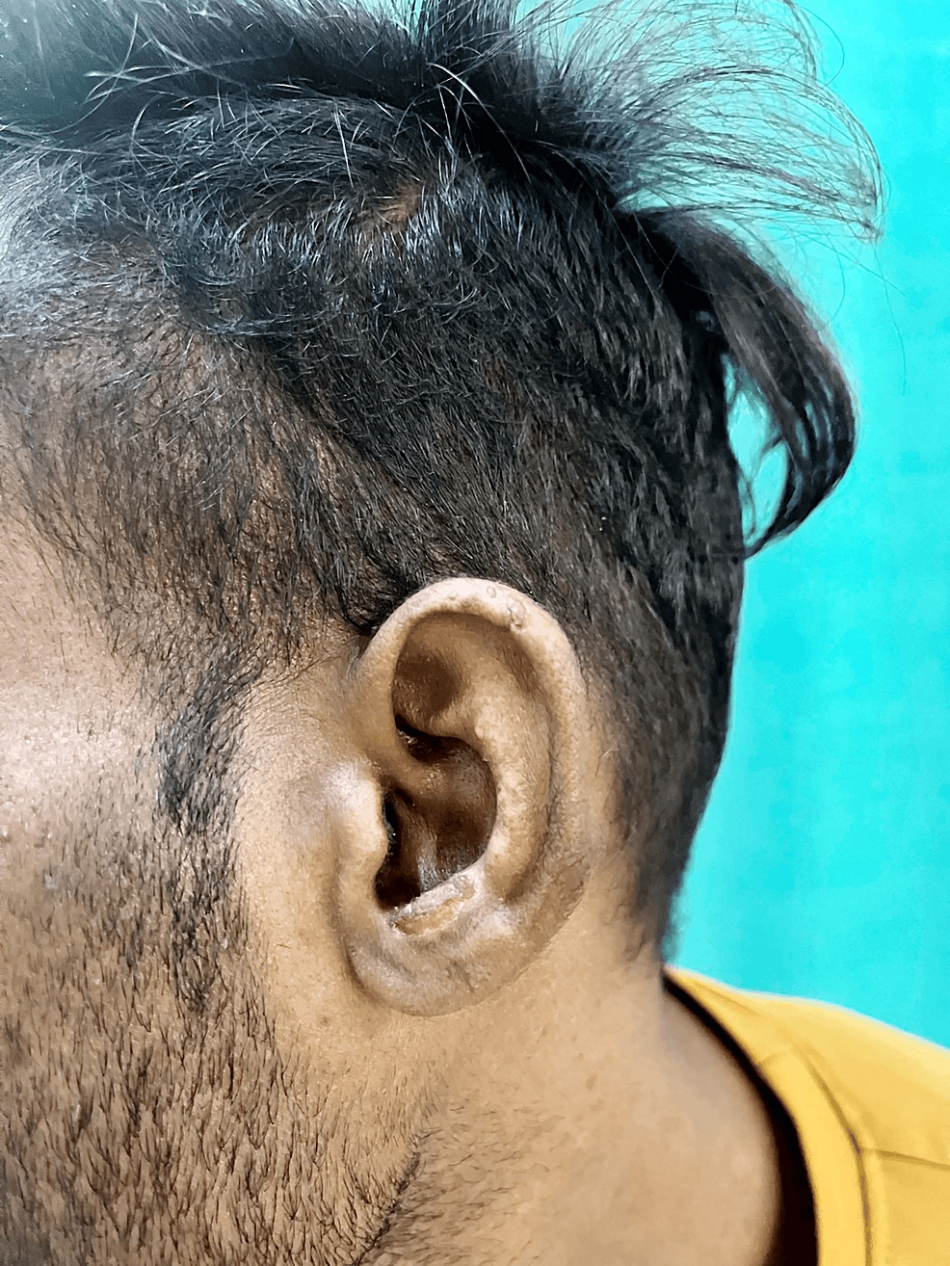
Erosions and scars over the pinna.

A 20-year-old male patient presented to our dermatology outpatient department with the chief complaints of multiple erosions with irregular scars on the face (Figure 1) and dorsum of bilateral hands (Figure 2) for one year. He gave a history of fluid-filled lesions over the same site, which were associated with itching and burning. These fluid-filled lesions would burst spontaneously over two to three days to form erosions (Figure 3), which would heal with scarring over two to three months. The lesions were associated with increased burning during sun exposure. On further inquiry, the patient reported a history of falls for which he took NSAIDs, oral diclofenac 50 mg, oral ibuprofen 400 mg, and paracetamol 325 mg, intermittently. There was no mucosal involvement.

**Figure 4:**
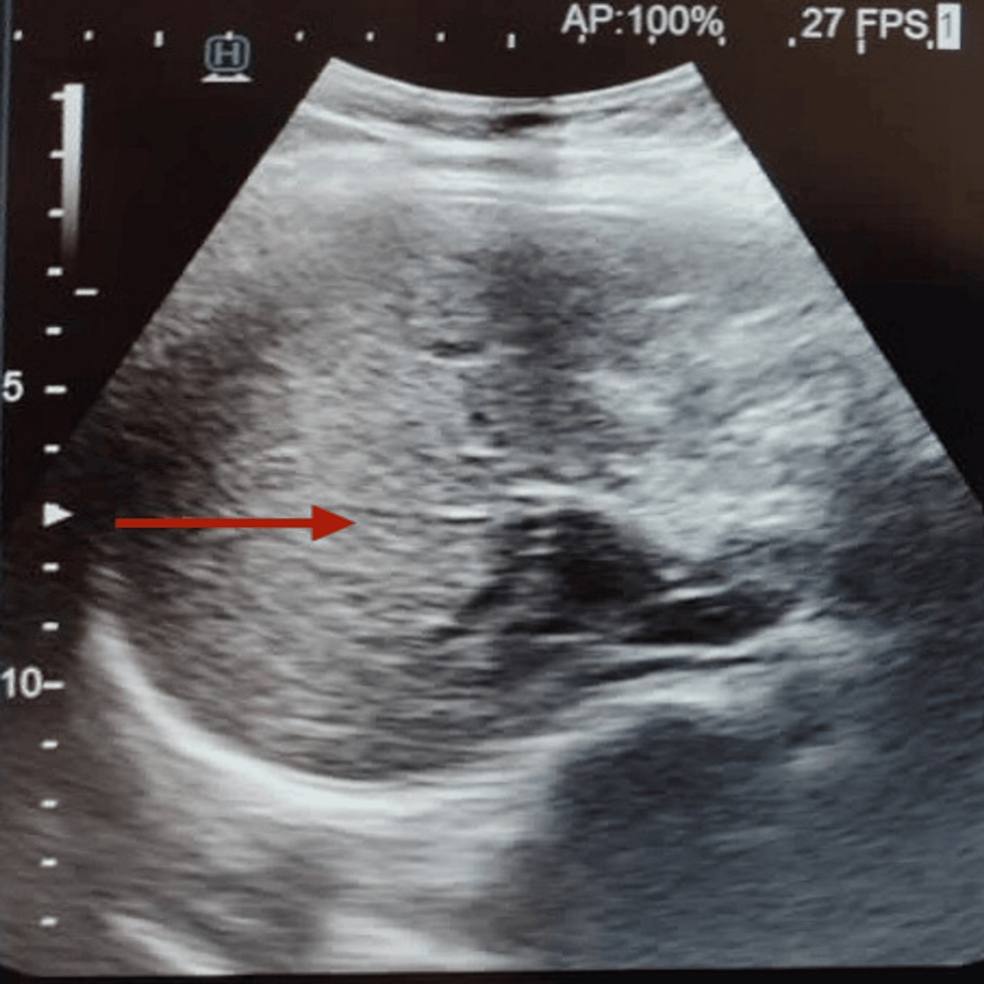
Ultrasonography of the liver with hyperechoic areas (depicted by the red arrow) suggestive of liver fibrosis.

**Figure 5:**
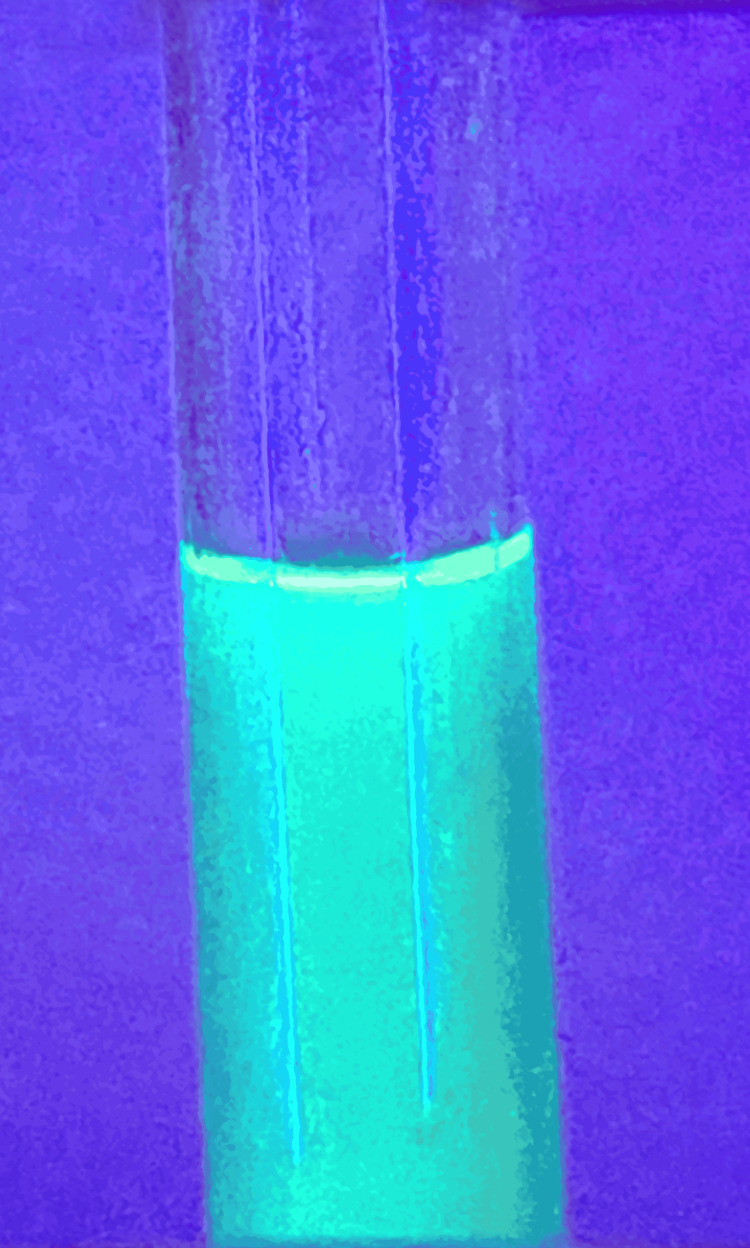
Wood’s lamp examination of the urine showing no fluorescence.

Systemic examination was within normal limits. The biochemical profile showed an elevation of alanine aminotransferase and aspartate aminotransferase, as shown in Table 1. Ultrasonography of the abdomen revealed altered echotexture of the liver, as shown in Figure 4. A gastroenterology opinion was sought given deranged liver enzymes, following which a fibrotouch scan was done, which revealed F3 fibrosis of the liver. Wood’s lamp examination of urine showed no fluorescence (Figure 5). Urine porphobilinogen was slightly elevated at 9.45 µmol/24 hours (average value <8.8 µmol).

**Table 1:** Laboratory values of the patient with reference values.

Laboratory parameters	Patient	Reference
Alanine transaminase	100 U/L	<50 U/L
Aspartate transaminase	83 U/L	17–59 U/L

Based on the clinical features, a diagnosis of pseudoporphyria was made, and the patient was started on a tablet of hydroxychloroquine sulfate 200 mg once daily along with strict sun protection and topical steroid (clobetasol propionate 0.05% cream). After one month of subsequent visits, the patient showed significant improvement.

## Discussion

Multiple causal agents for pseudoporphyria were reviewed by Green et al. [1]. These agents include drugs such as antibiotics, tanning beds, NSAIDs, and chronic renal failure/dialysis. They also covered many other factors, such as cyclosporine, 5-fluorouracil, and excessive Coca-Cola consumption. The study proposed that the action spectrum of UV light in pseudoporphyria would appear to be in the UVA range as pseudoporphyria is linked to photosensitizing medications, prolonged sun exposure, tanning beds, and psoralen and UVA therapy [1]. Garg et al. reported the case of a 30-year-old male who presented with multiple tense bullae and vesicles over his hands and feet. Chromatography revealed that both plasma and urine porphyrin were negative. As no apparent predisposing factor was detected, a further inquiry revealed increased sunlight exposure and consumption of country mallow, also known as “Khareti.” They suggested that consumption of country mallow and increased exposure to sunlight and pesticides could cause pseudoporphyria [2].

Quaiser et al. reported the case of a 49-year-old male with chronic kidney disease stage 3 with hypertension who was started on medications and presented with multiple pruritic vesicular lesions over bilateral hands. There have been cases of complete resolution in hemodialysis-associated pseudoporphyria following treatment with N-acetylcysteine, a glutathione precursor (800-1,200 mg p.o. daily for eight weeks). When a suspected photosensitizing drug is stopped in cases of drug-induced pseudoporphyria, improvement is typically observed within weeks to months (with an average of eight weeks), as seen in this case [3]. The case of a 76-year-old male patient with stage 4 chronic renal disease who was on furosemide persistently was reported by Pavanelli et al. [4]. The patient exhibited ulcerated lesions in his legs, accompanied by central hematic crust and peripheral erythema. Individuals with chronic kidney disease have a wide range of skin manifestations that are frequently undervalued because of their multifactorial nature and potential correlations with electrolyte imbalance, uremic material buildup, and comorbidities. Therefore, to make a diagnosis, a thorough laboratory and histopathological evaluation are essential [4].

Two cases of children receiving erythropoietin and peritoneal dialysis were documented by Harvey et al. [7]. Case 1 concerned a seven-year-old child receiving peritoneal dialysis for end-stage renal failure. She developed numerous vesicles across sun-exposed areas within two months of commencing erythropoietin. Another case was of a 12-year-old child who had glomerulonephritis-related chronic renal failure. Within two weeks of starting erythropoietin, he began to develop several fluid-filled lesions across sun-exposed areas [7]. Gyldenløve et al. [8] discussed the case of a 69-year-old paraplegic woman with a history of antiphospholipid syndrome and vitiligo who presented with tense bullae on both knees following sun exposure. The patient was receiving tolterodine, letrozole, and warfarin. Tolterodine was thought to be the drug of concern in this instance. The triggering agent was eliminated, and the patient was advised to avoid intensive sunlight [8].

In a case reported by Tremblay and Veilleux [9], the extensor surfaces of the hands and forearms of a 34-year-old woman undergoing hemodialysis showed blisters and atrophic scars. After three months, strict avoidance of direct sun exposure and regular application of a broad-spectrum SPF 60 sunscreen were unsuccessful in preventing more blistering. After starting therapy with 400 mg of N-acetylcysteine orally twice a day (powder dissolved in orange juice), clinical outcomes were notable in just three months. In vivo, intracellular glutathione production is enhanced by exogenous N-acetylcysteine treatment in conditions of elevated oxidative stress [9]. Cohen [10] reported prompt development of medication-associated pseudoporphyria (MAPS) in patients who received 5-fluorouracil, flutamide, and olaparib. On the other hand, individuals on tyrosine kinase inhibitors saw a four-month delay in the onset of MAPS-related skin lesions. Hence, the mechanism of pathogenesis for the development of MAPS is different based on the signaling pathways affected by tyrosine kinase inhibitors [10]. A summary of the case reports with underlying causes for pseudoporphyria is presented in Table 2.

**Table 2:** A summary of the case reports with underlying causes for pseudoporphyria.

Case number	Author(s)	The underlying cause for pseudoporphyria
1	Garg et al. [2]	Country mallow or “Khareti”
2	Pavanelli et al. [4]	Furosemide with chronic kidney disease stage 4
3	Harvey et al. [7]	Erythropoietin and peritoneal dialysis
4	Gyldenløve et al. [8]	Tolterodine
5	Tremblay and Veilleux [9]	Hemodialysis
6	Cohen [10]	5-fluorouracil, flutamide, and olaparib

## Conclusions

Pseudoporphyria is a rare and probably underdiagnosed clinical condition that various drugs, excessive sun exposure, and underlying diseases can cause. It is a bullous dermatosis in photo-distributed areas that share clinical and histological similarities with PCT without concomitant biochemical porphyrin abnormalities. The absence of fluorescence in pseudoporphyria is due to normal or near-normal urine porphyrins. Hence, knowing its particularities is essential for the differential diagnosis of skin lesions in patients with risk factors. Patients with chronic kidney disease may experience higher morbidity and mortality as a result of skin changes, which are often benign. As a result, its recognition is crucial for early diagnosis and appropriate treatment.

Drug-induced pseudoporphyria can occur due to various drugs that predominantly include NSAIDs, antibiotics, retinoids, diuretics, oral contraceptives, and kidney dialysis. In cases of drug-induced pseudoporphyria, the formation of phototoxic metabolites in genetically predisposed individuals leads to the formation of fluid-filled lesions. In these cases, rechallenge with the offending drug to produce symptom relapse has been proposed to help confirm this diagnosis of exclusion.
